# Differential Regulation of Ferritin Subunits and Iron Transport Proteins: An Effect of Targeted Hepatic X-Irradiation

**DOI:** 10.1155/2013/353106

**Published:** 2013-12-12

**Authors:** Naila Naz, Shakil Ahmad, Silke Cameron, Federico Moriconi, Margret Rave-Fränk, Hans Christiansen, Clemens Friedrich Hess, Giuliano Ramadori, Ihtzaz A. Malik

**Affiliations:** ^1^Department of Gastroenterology and Endocrinology, University Medical Center, Georg-August University, Robert-Koch Straße 40, 37075 Göttingen, Germany; ^2^Department of Radiation Therapy and Radiooncology, University Medical Center, Georg-August University, Robert-Koch-Straße 40, 37075 Göttingen, Germany; ^3^Department of Radiation Oncology, Medizinische Hochschule Hannover, Carl-Neuberg Straße, 30625 Hannover, Germany

## Abstract

The current study aimed to investigate radiation-induced regulation of iron proteins including ferritin subunits in rats. Rat livers were selectively irradiated *in vivo* at 25 Gy. This dose can be used to model radiation effects to the liver without inducing overt radiation-induced liver disease. Sham-irradiated rats served as controls. Isolated hepatocytes were irradiated at 8 Gy. Ferritin light polypeptide (FTL) was detectable in the serum of sham-irradiated rats with an increase after irradiation. Liver irradiation increased hepatic protein expression of both ferritin subunits. A rather early increase (3 h) was observed for hepatic TfR1 and Fpn-1 followed by a decrease at 12 h. The increase in TfR2 persisted over the observed time. Parallel to the elevation of AST levels, a significant increase (24 h) in hepatic iron content was measured. Complete blood count analysis showed a significant decrease in leukocyte number with an early increase in neutrophil granulocytes and a decrease in lymphocytes. *In vitro*, a significant increase in ferritin subunits at mRNA level was detected after irradiation which was further induced with a combination treatment of irradiation and acute phase cytokine. Irradiation can directly alter the expression of ferritin subunits and this response can be strongly influenced by radiation-induced proinflammatory cytokines. FTL can be used as a serum marker for early phase radiation-induced liver damage.

## 1. Introduction

Therapeutic radiation causes both acute and chronic toxicity in normal tissue [1]. Indeed, radiation-induced liver disease (RILD) is a serious clinical complication [2], due mainly to vessel damage. Radiation-induced inflammation is known to be mediated by cytokines [3] probably through activation of their transcription factors. These signalling cascades result in an increase in the plasma levels of a number of positive acute phase proteins (APPs) [4] and change in gene expression of several iron regulatory proteins [3, 5].

In the clinical setting, when radiation is applied to the entire liver, RT doses of 30 to 33 Gy carry a risk of about 5% of radiation-induced liver disease [6]. Most solid tumors require total RT doses of at least 60 Gy [7]. With the advent of stereotactically guided radiotherapy (SBRT), single dose radiation for hepatic metastases was possible with a medium dose of 24 Gy; range 17–30 Gy [8]. For fractionated radiation of HCC, total dose of 24–60 Gy with daily doses of 2.3–2.5 Gy has been used [9, 10]. Similar doses can be given to hepatic metastases [11]. With an increasing Child-Pugh score and decreasing liver function, total doses were reduced to 35 Gy in Child B patients [12]. Our model of 25 Gy single dose irradiation is a well-understood simplified model to study radiation-induced liver damage [3, 13–18]. It is minimally toxic at a high single dose. It can further be compared with other models of liver damage [16, 17, 19]. Studies on fractionated liver injury are on their way [20].

In the current study, we focused on the differential regulation of ferritin subunits under single dose 25 Gy liver irradiation. Iron is essential for metabolic processes. A large group of iron regulatory proteins controls iron homeostasis such as transferrin (Tf) [21], transferrin-receptors (TfRs) [22], and ferroportin-1 (Fpn-1) [23]. Within the cell, iron is mainly stored as ferritin [24]. In humans, ferritin is composed of two subunits: ferritin L and ferritin H. Both subunits are highly conserved [25], nevertheless, genetically separated [26, 27], and maintain distinct functions [28]. Both subunits are differentially and independently regulated on both, transcriptional and posttranscriptional level [28]. Serum ferritin levels are widely used in clinical settings and have become a part of the routine assessment of human body iron stores [29, 30]. It has, however, become increasingly evident that several clinical conditions can be associated with elevated serum ferritin levels in the absence of iron overload [28, 31], while the identity of serum ferritin is not clear and its source is still a matter of debate [32].

The mechanism of iron uptake is partially known in other models but poorly understood in liver after irradiation. Single dose X-irradiation reduced the serum iron and can induce changes in hepatic expression of iron regulatory genes at mRNA level [3]. The aim of our prospective study is to extend the previous knowledge and monitor the changes at protein and mRNA level in hepatic iron transport (TfRs and Fpn-1) and storage (ferritin) proteins after targeted liver X-irradiation. Furthermore, we aimed to investigate the effect of X-irradiation as regulator of ferritin subunits in the presence or absence of acute phase cytokines in isolated cultured hepatocytes.

## 2. Methods

### 2.1. Animal Model

A rat model for targeted liver X-irradiation was established by CT planned single organ X-irradiation as described before [3]. Rats were kept and sacrificed under standard conditions approved by institution's guidelines, as designated earlier [33]. Treated and sham-irradiated controls were sacrificed at 1, 3, 6, 12, 24, and 48 h after irradiation. Blood was collected to obtain serum. Liver tissues were taken carefully, rinsed with 0.9% NaCl, snap frozen in liquid nitrogen, and preserved at −80°C for further use.

### 2.2. Isolation and Culture of Rat Hepatocytes

Hepatocytes were isolated from normal animals according to a protocol described previously [34]. The purity of the isolated cell population was determined by phase contrast microscopy and by immunocytochemistry using antibodies against laminin or GFAP to identify stellate cells (both from Sigma, Deisenhofen, Germany) or ED1 and ED2 (gift from C. Dijkstra) for macrophages. Dulbecco's modified Eagle's medium (DMEM) (Biochrom, Berlin, Germany) was supplemented with 10% fetal calf serum (FCS) (PAA, Cölbe, Germany), 1 nM insulin (Roche, Mannheim, Germany), and 100 nM dexamethasone (Sigma, Munich Germany). After isolation, the hepatocytes were either irradiated (8 Gy) with 6 mV Photons using Variance Clinac 600C accelerator [17] or irradiated and additionally exposed to 500 ng of recombinant interleukin (IL) 1*β*, IL-6, or TNF-*α* (PeproTech, Rocky Hill, NJ) in 3 mL cell culture supernatants immediately before irradiation.

### 2.3. Preparation of Tissue Lysates for Total Protein Isolation and Western Blot Analysis

About 50 mg of frozen tissue was homogenized with an Ultra-Turrax TP 18/10 three times for 10 s each. Lysis buffer contained 50 mM Tris-HCl buffer (pH 7.4), 150 mM sodium chloride, 1 mM ethylenediaminetetraacetic acid, 1% Triton X-100, 1 mM phenylmethanesulfonyl fluoride, 1 mM benzamidine, 1 mg/mL leupeptin, 10 mM chymostatin, 1 mg/mL antipain, and 1 mg/mL pepstatin A. The entire procedure was carried out at 4°C. Crude homogenates were passed five times through a 22 G needle attached to a syringe, centrifuged for 5 min at 10,000 g and 4°C. The protein concentration was determined in supernatants using the Bradford protein assay reagent (Pierce, Germany). Aliquots of the homogenates were stored at −20°C until further use for western blot analysis.

50 *μ*g of protein from tissue and serum was applied per well for electrophoresis using NuPAGE-SDS-PAGE (4–12% Bis-Tris Gel; Invitrogen, USA) under reducing conditions [35]. After electrophoresis; the proteins were transferred to Hybond-enhanced chemiluminescence (ECL) nitrocellulose membranes [36]. Western blot was performed utilizing FTL (Abcam and Santa Cruz) in 1 : 1000 and 1 : 100 dilution, respectively. FTH (Santa Cruz, Life Sciences Bio) in 1 : 100 and 1 : 250 dilution, respectively, TfR-1 (Invitrogen) in 1 : 1000 dilution, TfR-2 (Abcam) in 1 : 500 dilution, Fpn-1 (Pro-science) in 1 : 500 dilution, *β*-actin (Sigma) in 1 : 4000 dilution. Immunodetection was performed utilizing ECL reagent.

### 2.4. RNA Isolation and Quantitative Real-Time PCR

Total RNA was isolated from cultured cells by means of guanidine isothiocyanate extraction, caesium chloride density-gradient ultracentrifugation, and ethanol precipitation according to a previously described method [37], with some modifications as described elsewhere [38]. The cDNA was generated by reverse transcription of 1 *μ*g of total RNA using 100 nM of dNTPs, 50 pM of primer oligo dT15, 200 U of moloney murine leukaemia virus reverse transcriptase (M-MLV RT), 16 U of protector RNase inhibitor in RT buffer, and 2.5 *μ*L of 0.1 M DTT; real-time PCR was performed using an ABI prism 7000 sequence detection system as described elsewhere [17]. The primer sequences used for RT-PCR are FTL 5-3 AACCACCTGACCAACCTCGCTA 5′-3′ TCAGAGTGAGGCGCTCAAAGAG, FTH 5-3 GCC CTG AAG AAC TTT GCC AAAT, 5′-3′TGCAGGAAGATTCGTCCACCT, UBC5-3 CACCAAGAAGGTCAAACAGGAA 5′-3′ AAGACACCTCCCCATCAAACC, and *β*-actin 5-3 TGT CAC CAA CTG GGA CGA TA 5′-3′ AAC ACA GCC TGG ATG GCT AC. *β*-actin and ubiquitin C were used as housekeeping genes. The results were normalized to the housekeeping gene and fold change expression was calculated using threshold cycle (*C*_*t*_) values.

### 2.5. Measurement of Circulating Aspartate Aminotransferase (AST) and Complete Blood Count (CBC)

At time points ranging from 1 to 48 hours after targeted hepatic X-irradiation, blood samples from the inferior vena cava were collected from sham-irradiated and irradiated rats and used for AST measurement using analysis kits (DiaSys, Germany) as instructed. The number of leukocytes in the blood of irradiated and sham-irradiated rats was determined by routine measurement.

### 2.6. Tissue Iron Level

Hepatic iron levels were measured utilizing a colorimetric ferrozine based assay [39]. To measure the iron concentration, tissue homogenates were prepared as reported previously [5].

### 2.7. Statistical Analysis

The data was analysed using Prism Graph pad 4 software (San Diego, USA). All experimental errors are shown as SEM. Statistical significance was calculated by one-way ANOVA and Dunnett post hoc test. Significance was accepted at *P* < 0.05.

## 3. Results

### 3.1. Changes in Iron Storage and Transport Proteins in Serum and Liver after Irradiation

**Figure 1:**
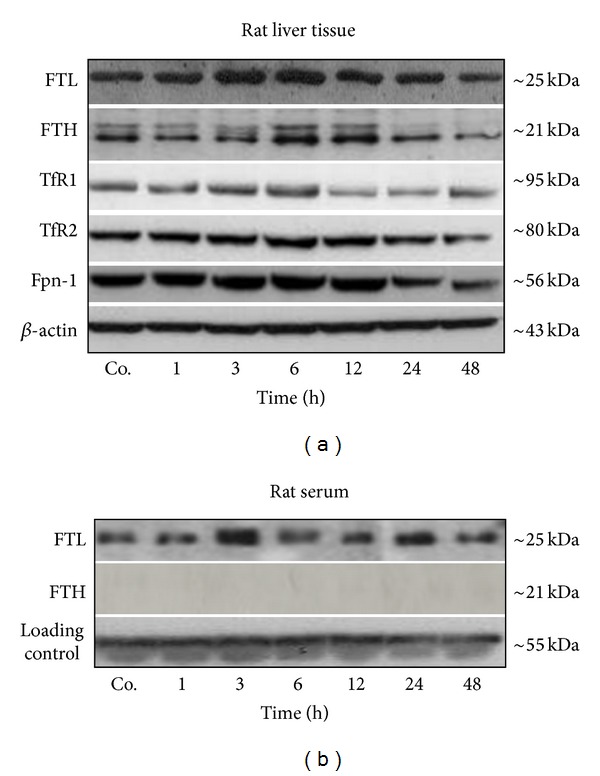
(a) Western blot analysis of iron storage (FTL and FTH) and iron transport protein (TfR1, TfR2 and Fpn-1) in rat liver of control and irradiated animals. (b) Western blot analysis of iron storage (FTL and FTH) proteins from serum total protein. *β*-actin was used as a loading control in liver while, in serum loading control represents an internal loading control (*∼*55 kDa). Results are representative of three experiments.

The protein amount of both, hepatic FTL and FTH was found to be elevated after single dose liver X-irradiation. By means of western blot FTL protein level was found to increase early (3 h) and remained above the control level until 24 h, whereas hepatic FTH revealed a maximum increase at 6–12 h after liver irradiation (Figure 1(a)). An early (3 h) and slight increase was detected in hepatic iron import (TfR1 and TfR2) and export proteins (Fpn-1). In contrast, a decrease in hepatic TfR1 and Fpn-1 protein amount was evident at later time points. The minimum protein level of TfR1 was found at 12 h and for Fpn-1 at 24 h after rat liver irradiation (Figure 1(a)).

A marked constitutive expression of FTL was detected in the serum of sham-irradiated controls and irradiated rats, while FTH was undetectable at any time point by Western blotting. Moreover, the serum protein level of FTL was found to increase early (3 h) after irradiation (Figure 1(b)).

### 3.2. Changes in Circulating AST and Hepatic Iron Level after Irradiation

**Figure 2:**
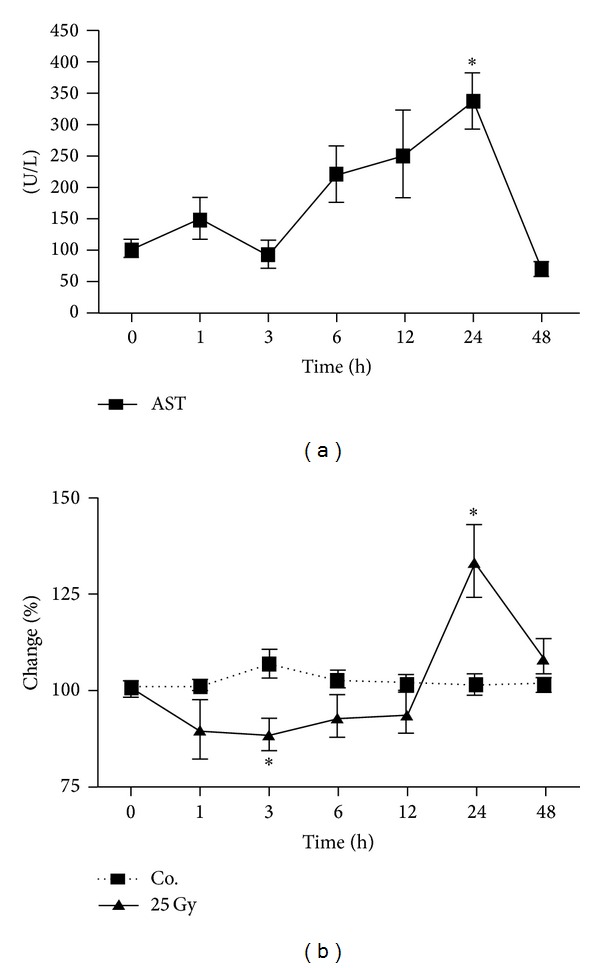
(a) Changes of circulating AST levels after targeted hepatic irradiation. (b) Changes in hepatic iron content after single dose liver irradiation. Iron levels were determined by ferrozine-based assay (Co. control). Results represent mean values ± SEM (*P < 0.05 analyzed by one-way ANOVA; *n* = 3).

Serum activity of AST was found to be elevated early after irradiation and reached a peak at 24 h. This increase was found to be statistically significant (Figure 2(a)). An early decrease in iron level followed by a significant increase at 24 h was measureable in the liver tissue after irradiation as compared to the controls (Figure 2(b)).

### 3.3. Changes in Circulating Leukocyte Number after Irradiation

**Figure 3:**
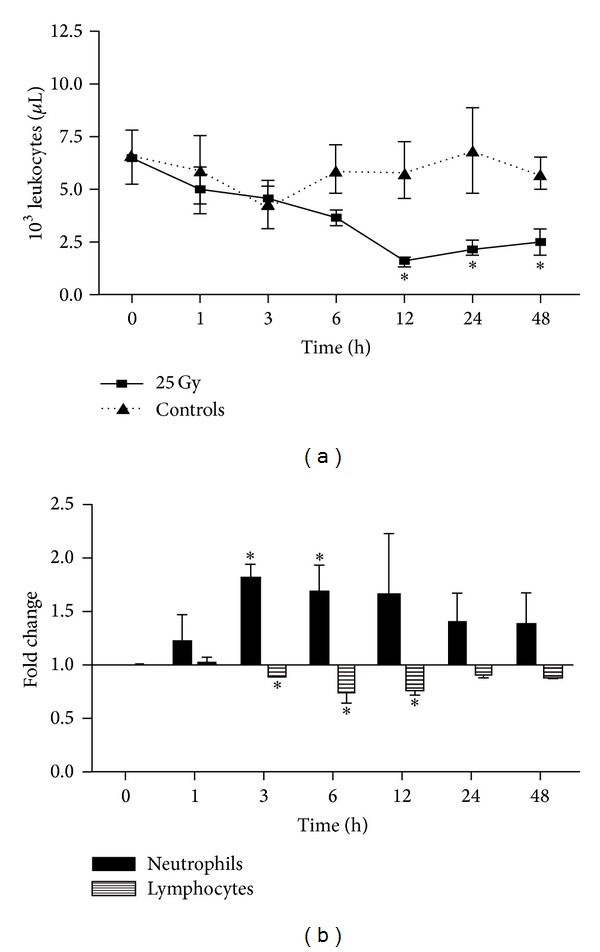
Changes in complete blood picture (CBC) after single dose liver irradiation. (a) Total leukocytes number. (b) Fold change in numbers of neutrophils and lymphocytes after targeted hepatic irradiation. Results represent mean values ± SEM (*P < 0.05 analyzed by one-way ANOVA; *n* = 3).

Complete blood count (CBC) analysis showed a decrease in total leukocyte count after liver irradiation. A rapid decrease (1 h) in total leukocytes number was observed with a minimum at 12 h. The leukocyte count remained significantly lower than in sham-irradiated controls throughout the course of study (Figure 3(a)).

Differential CBC revealed that the major cell population, which was decreased after liver irradiation, was lymphocytes. A significant reduction was observed after 3 h with a minimum at 12 h (approx. 30%) after irradiation. Furthermore, the numbers of lymphocytes remained below the control level until 48 h. In contrast, a significant increase in neutrophils was detected with a maximum (1.82 ± 0.13-fold) at 3 h (Figure 3(b)). However, no significant change in blood monocyte and erythrocyte (data no shown) number was found at any time after liver irradiation.

### 3.4. Modulation of FTL and FTH Protein Gene Expression in Isolated Rat Hepatocytes after Irradiation

**Figure 4:**
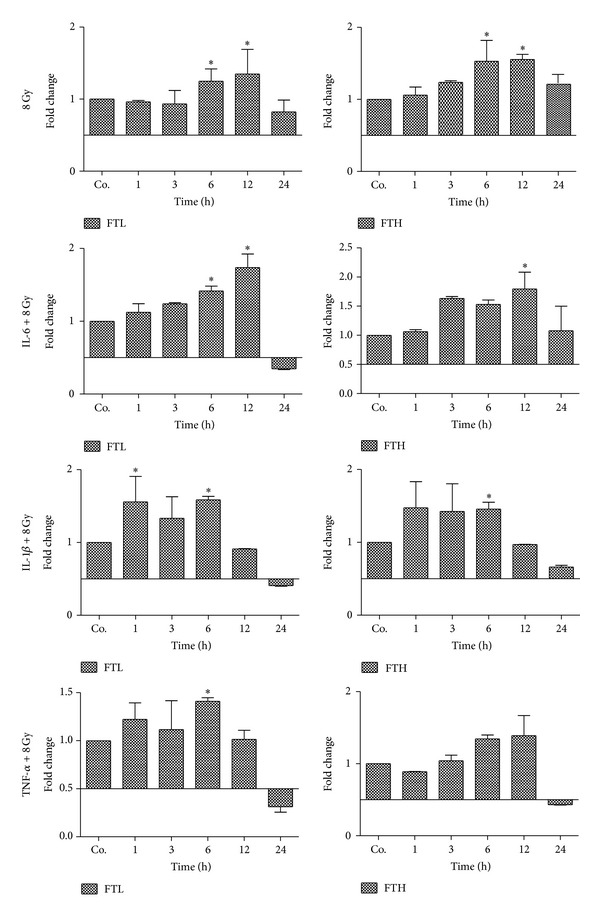
qRT-PCR analysis of total RNA from isolated rat hepatocytes treated with irradiation 8 Gy, alone or in combination with major acute phase cytokines (IL-6, IL-1*β* and TNF-*α*). Data are shown as fold changes in mRNA expression of iron storage proteins (FTL and FTH) at various time points relative to untreated controls for each time point. qRT-PCR was normalized by using two housekeeping genes: *β*-actin and ubiquitin C. Results represent means ± SEM of three experiments; *P < 0.05, *n* = 3.

FTL and FTH mRNA level in isolated rat hepatocytes significantly increased upon radiation exposure (8 Gy) alone or in combination with major acute phase cytokines (IL-1*β*, IL-6, and TNF-*α*) compared with untreated controls. Irradiation exposure of hepatocytes led to an increase in mRNA expression of both ferritin subunits throughout the study with a maximum at 6–12 h. However, among the combination treatments, the most pronounced increase was observed after treatment with IL-6 and irradiation with a maximum at 12 h. IL-1*β* had an early (1 h) effect on FTL and FTH gene expression when combined with irradiation treatment (Figure 4).

## 4. Discussion 

In this work, a brisk increase of FTL in the serum was detected after single dose liver irradiation. FTH, however, was not detected in the serum, neither of sham-irradiated controls nor of irradiated rats. In addition, hepatic protein expression of both genes was found to be elevated after irradiation. Similar to ferritin subunits, a mild early increase in iron import (TfR1 and TfR2) and iron export (Fpn-1) protein expression was observed, whereas an evident decrease in TfR1 and Fpn-1 at later time points (24 h) was found after irradiation.

Moreover, an increase in serum AST levels was detected in parallel to the increase in hepatic tissue iron levels. Complete blood count (CBC) analysis showed a significant decrease in leukocyte count over time, with an early increase in granulocyte count and a decrease in lymphocyte count after liver irradiation.

Ferritin is composed of two subunits: ferritin L and ferritin H; however, previously ferritin was mostly studied as a single unit and role of its subunits was poorly described. Both subunits are highly conserved [25], nevertheless, genetically separated [26, 27], and maintain distinct functions [28]. Several observations reported that ferritin subunits (FTH and FTL) are iron storage proteins and their amount could only be modified by changes in iron status [40–42]. However, others and we reported that change in ferritin-subunits is not only due to the increase in hepatic iron concentration, but it could also be due to the direct effect of different inflammatory mediators such as acute-phase cytokines [33, 43–45]. Likewise our current study extended our previous knowledge where we showed an upregulation of proinflammatory cytokines in the same model [3] suggesting that a change in gene expression of ferritin subunits is also induced directly by irradiation or/and irradiation-induced cytokines. In our current study, we showed changes in ferritin subunits induced directly by irradiation. This is an important aspect, as serum ferritin has been described as a risk factor for veno-occlusive disease [46]. It should, hence, be monitored under liver irradiation. In this context it has been shown that iron chelation may reduce the risk of hepatic veno-occlusive disease prior to high dose chemotherapy and auologous stem cell transplantation [47]. Moreover, hemostasis as well as cytokine and chemokine regulation is involved in veno-occlusive liver disease [48]. Both these factors are affected by radiation treatment [15, 49, 50]. The current study supports these observations.

In addition, by using different detection methods (ELISA and western blot), we previously showed in different model that FTL serves as a secretory protein in a model of sterile abscess (acute phase response), where the liver responded to circulating acute phase cytokines produced at the site of injury [33]. Accordingly, in the current study, targeted hepatic X-irradiation resulted in a release of liver FTL into the serum. It indicates that FTL not only shares “the iron storage” function but also behaves as a secretory protein in the liver. This can also be true in the current study where an increase in serum FTL levels and hepatic FTL expression was observed after irradiation, suggesting that stress to the liver (in the present study caused by liver irradiation) induced hepatic FTL expression which could be responsible for an increase in serum FTL.

TfR1 is thought to be inversely regulated by the cellular iron status [51]. This is in accordance with our findings in rat liver after X-irradiation. An increase in the iron import proteins and later reduced expression of the iron export protein Fpn-1—in parallel to increased hepatic iron levels—could suggest a transient iron retention within the hepatocyte to fulfil the requirements of damage or stress conditions (increased AST level) caused by irradiation.

Another aspect of the current study was the observation of leucopenia mainly with low lymphocyte numbers after irradiation. This could explain the only mild inflammation with marginal hepatic increase in neutrophil granulocytes in our model [17]. Radiation could have reduced the number of leukocytes available for mounting an inflammation. A link between lower iron levels and decrease in leukocyte number have already been described [52, 53]. A study showed a lower lymphocyte number in patients with iron deficiency anemia (IDA) which could also be true in our studied model [52].

Clinically, regulation of iron metabolism could be an underestimated response in radiotherapy. Further understanding of iron metabolism before and during radiotherapy could help to understand irradiation-induced hepatocellular damage/fibrosis and the impact of reduced iron levels on immune responses in patients with iron deficiency anemia. A strong correlation between radiation-induced cytokines and iron regulatory proteins has been found in patients with prostate cancer after radiotherapy [14]. However, further prospective clinical studies need to be performed to correlate iron metabolism dynamics with the clinical course of patients developing irradiation-induced problems. This would help to understand whether a change in iron or iron regulatory proteins could predict onset of symptoms.

Taken together, our data suggest that ferritin subunits are influenced not only by the iron status but also by cytokines as well as irradiation directly. This hypothesis was further confirmed by our *in vitro* experiments (isolated cultured hepatocytes) where a differentially regulated expression of ferritin subunits was found in hepatocytes after exposure to irradiation together with cytokines. Furthermore, FTL can be a potential biomarker for early phase radiation-induced liver damage.
